# Leptospirosis in Malaysia: current status, insights, and future prospects

**DOI:** 10.1186/s40101-023-00347-y

**Published:** 2023-12-12

**Authors:** Noraini Philip, Kamruddin Ahmed

**Affiliations:** 1 School of Biological Sciences, Universiti Sains Malaysia, Penang, Malaysia; 2 Department of Pathology and Microbiology, Faculty of Medicine and Health Sciences, University Malaysia Sabah, Kota Kinabalu, Malaysia; 3 Borneo Medical and Health Research Centre, Faculty of Medicine and Health Sciences, Universiti Malaysia Sabah, Kota Kinabalu, Malaysia; 4 Research Center for Global and Local Infectious Diseases, Oita University, Oita, Japan

**Keywords:** Leptospirosis, Epidemiology, Risk factors, Serovars, Malaysia

## Abstract

Among zoonotic infections, leptospirosis has a worldwide distribution and high prevalence in tropical regions. It has a broad clinical presentation from mild to severe, life-threatening infection. Leptospires, the etiological agent of leptospirosis, are found in varied ecological niches and animal species, providing a significant source of human infection. This review aims to provide the current status of leptospirosis in Malaysia and the direction for future studies. The literature search for this review was performed using PubMed, Web of Sciences, and Google Scholar databases. The incidence of leptospirosis in Malaysia from 2004 to 2020 varied; however, a large number of cases occurred during floods. *Leptospira* has been isolated from wild and domestic animals as well as from the environment; among them, several novel species have been identified. In Malaysia, leptospirosis infection and death were mostly associated with recreational and non-recreational water activities. Despite the endemicity of leptospirosis, the public’s knowledge, attitude, and practice level are relatively low in this country. More studies are needed in Malaysia to explore the extent of leptospirosis in different settings and locations.

**Supplementary Information:**

The online version contains supplementary material available at 10.1186/s40101-023-00347-y.

## Background

Leptospirosis is a zoonotic infection; the risk factors of this infection are associated with human activities and the environment [1]. It is widely distributed, infecting more than one million people worldwide and causing 60,000 deaths yearly [2]. A helical and highly motile spirochete belonging to the genus *Leptospira* causes leptospirosis. Leptospires in nature are maintained by chronic carrier hosts, primarily rodents, in their renal tubules and excreted into the environment through their urine. Human infection results from direct contact with the infected reservoir animals or indirect exposure to contaminated environments. Leptospires enter the hosts through cuts and bruises on skin and mucus membranes such as the conjunctival, oral, or genital surfaces [3]. Once inside the hosts, the leptospires are disseminated by hematogenous routes into many organs, mainly the kidneys, liver, and lungs. Humans infected with leptospires typically manifest a broad clinical presentation, ranging from asymptomatic [4] or mild to severe and life-threatening infection. Multi-organ injuries and pulmonary hemorrhage characterize the severity. Its progression from mild to severe is rapid, as shown in human [5, 6] and animal studies [7, 8]. Severe leptospirosis has been associated with pathogen virulence, host susceptibility, and epidemiological conditions [3].

Leptospirosis is diagnosed by microscopic agglutination test (MAT), and polymerase chain reaction (PCR) targeting genes specific for pathogenic leptospires. The former method requires an understanding of locally circulating serovars. *Leptospira* is detected in broad ranges of animal reservoirs and in various ecological niches. The knowledge of ecological niche harboring leptospires, as well as their species and serovar distribution, is critical not only for implementing prevention and control policies but also for diagnosing leptospirosis.

The burden of leptospirosis is mainly in countries with humid subtropical and tropical climates such as South America, Southern Asia, and Southeast Asia. Malaysia is located in Southeast Asia in the north of the equator. It is composed of two noncontiguous regions: Peninsular Malaysia (Semanjung Malaysia) or West Malaysia (Malaysia Barat), which is located in the Malay Peninsula, and East Malaysia (Malaysia Timur), which is on Borneo Island, contains two states, Sabah and Sarawak. Peninsular and East Malaysia lie in the same tropical latitude and are affected by similar airstreams. From November to March, Malaysia is blessed with heavy rainfall and humidity during the northeast monsoon season. Leptospirosis in Malaysia has a long history, reaching almost a century.

Ten years after *Leptospira* was discovered in Japan [9], Fletcher first reported leptospirosis in Malaysia in 1925 among rubber plantation workers and people residing in rural areas [10]. Fletcher also identified two *Leptospira interrogans* serovars—namely, Icterohaemorrhagiae and Hebdomadis—isolated from humans, rats, and dogs [9]. Leptospires were also detected in water supply, streams, and ponds; however, their serovars remained unidentified. After the discovery of leptospires in Malaysia, studies were performed focusing on the military [11–13]. The prevalence of leptospirosis among military personnel ranged from 4.6 to 34.7%, including two fatal cases which were detected in 1969 and 1978 [13]. Several studies also reported that workers in rubber and oil palm plantations, hospitals, sewage, and town cleanings were at high risk of infection [14–17]. Several outbreaks were reported from Sarawak state among those involved in cave explorations [18, 19] and chamber visitations [20]. Leptospires were also detected in domestic animals such as cattle, buffaloes, and pigs with *L. interrogans*, Unipertama, Canicola, Australis, Javanica, Ballum, Pomona, Hardjo, Sejroe, and Tarassovi as the infecting *Leptospira* species and serovars [21–23]. In humans, Malaya [24], Abramis, Biggis, Birkini, Coxi, Fugis, Gurungi, Hemolytica, Hamptoni, Mooris, Ricardi, Smithi, and Sumneri were isolated from leptospirosis patients [25]. These earlier reports showed that occupations determined the occurrence of leptospirosis. The present review aims to describe the current status of leptospirosis in Malaysia, surveyed from 2000 to 2021, define research gaps, and update the importance of developing prevention measures.

## Literature search and ethics

The published literature related to leptospirosis in Malaysia was searched in PubMed, Web of Sciences, and Google Scholar databases. The search terms utilized were “Leptospirosis”, “*Leptospira*,” “Prevalence,” “Seroprevalence,” “Human,” and “Animal” with no restriction on language or publication date. Non-indexed local journals, bulletins, newspapers, clinical cases, and data from the Ministry of Health Malaysia were included in our results and discussion. Additional papers were identified from reference lists of retrieved articles to find appropriate studies that might not have been identified during the preliminary search. We reviewed the published literature with anonymized data; thus, the study does not require any bioethical approval.

## Leptospirosis in Malaysia

**Fig. 1:**
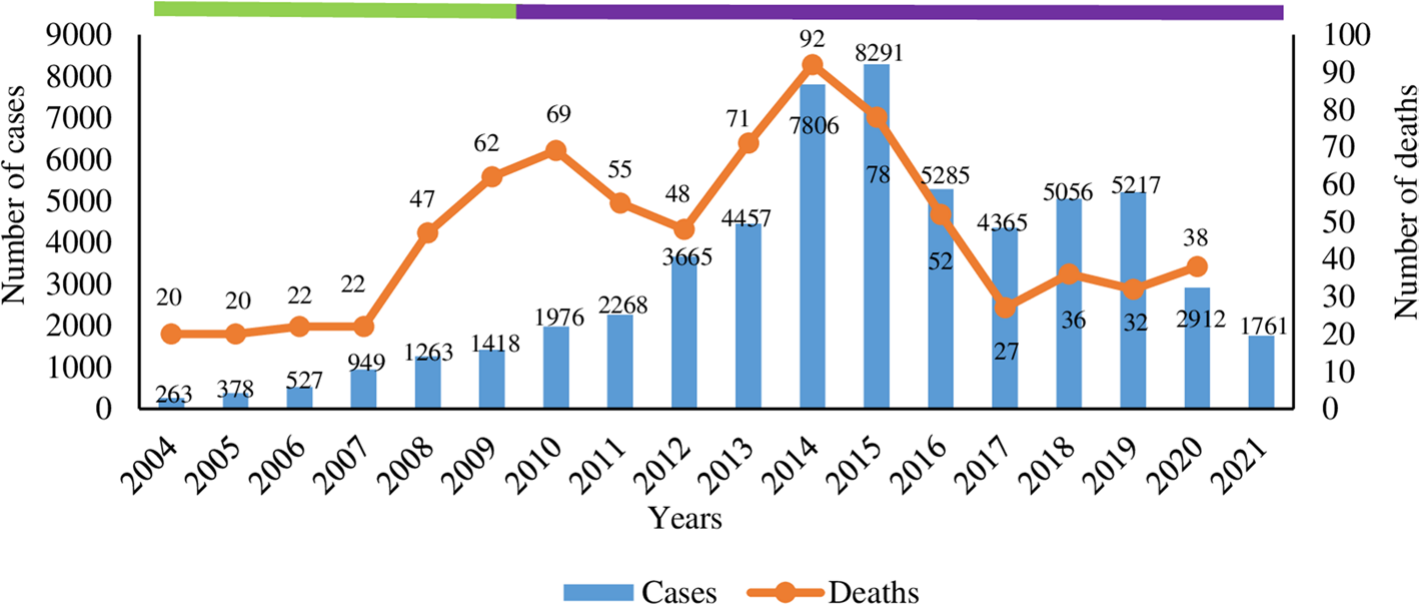
Leptospirosis cases and deaths in Malaysia from 2004 to 2021. The yearly distribution of leptospirosis cases and deaths in Malaysia from 2004 to 2021. The number of cases from 2004 to 2009 (green horizontal bar) does not represent the actual number of cases as leptospirosis disease notification only became mandatory in 2010 (purple horizontal bar). The cases increased gradually from 2010 to 2013 and then exponentially from 2013 to 2015. Then, it decreased in 2016 and 2017, increased in 2018 and 2019, and decreased again in 2020 and 2021. The incidence of leptospirosis cases remained similar from 2004 to 2007 and sharply increased in 2008 and peaked in 2010, declined till 2012 and peaked again in 2014, gradually declined till 2017, and increased a little in 2018 and continued a similar trend. Data on leptospirosis incidence in 2021 was not available. Source of data: Ministry of Health Malaysia, Annual Report 2010–2021. Estimated number of cases (2004–2009); Purple bar: The recorded number of cases (2010–2021)

Leptospirosis is endemic in Malaysia and is the third most fatal infection after dengue and malaria [26]. In 2010, leptospirosis was gazetted as a notifiable disease in this country [27]. Since then, the number of reported cases and their mortality have been recorded. A higher number of leptospirosis was recorded from 2010 to 2019, with the peak of leptospirosis cases and deaths in 2014 and 2015, respectively (Fig. 1). In 2020 and 2021, the incidence of leptospirosis decreased, and a similar trend was also observed in European countries during the same period [28]. This decline could be attributed to the changes in population behaviors, such as reduced outdoor and recreational activities and the closure of schools in compliance with the movement control order (MCO) due to COVID-19 [29]. The incidence and case fatality rates (CFR) of leptospirosis in Malaysia between 2010 and 2020 were 8.63–17.2 and 0.6–2.4%/100,000 populations, respectively [29–39]. In 2020, although the incidence rate was low, the CFR was high compared to the previous year (2020: 1.3%, 2019: 0.6%). Leptospirosis outbreaks were also reported and mainly occurred in residential areas. More than half of the infected patients were men in the age group of 20–60 years [27, 29–39].

**Fig. 2:**
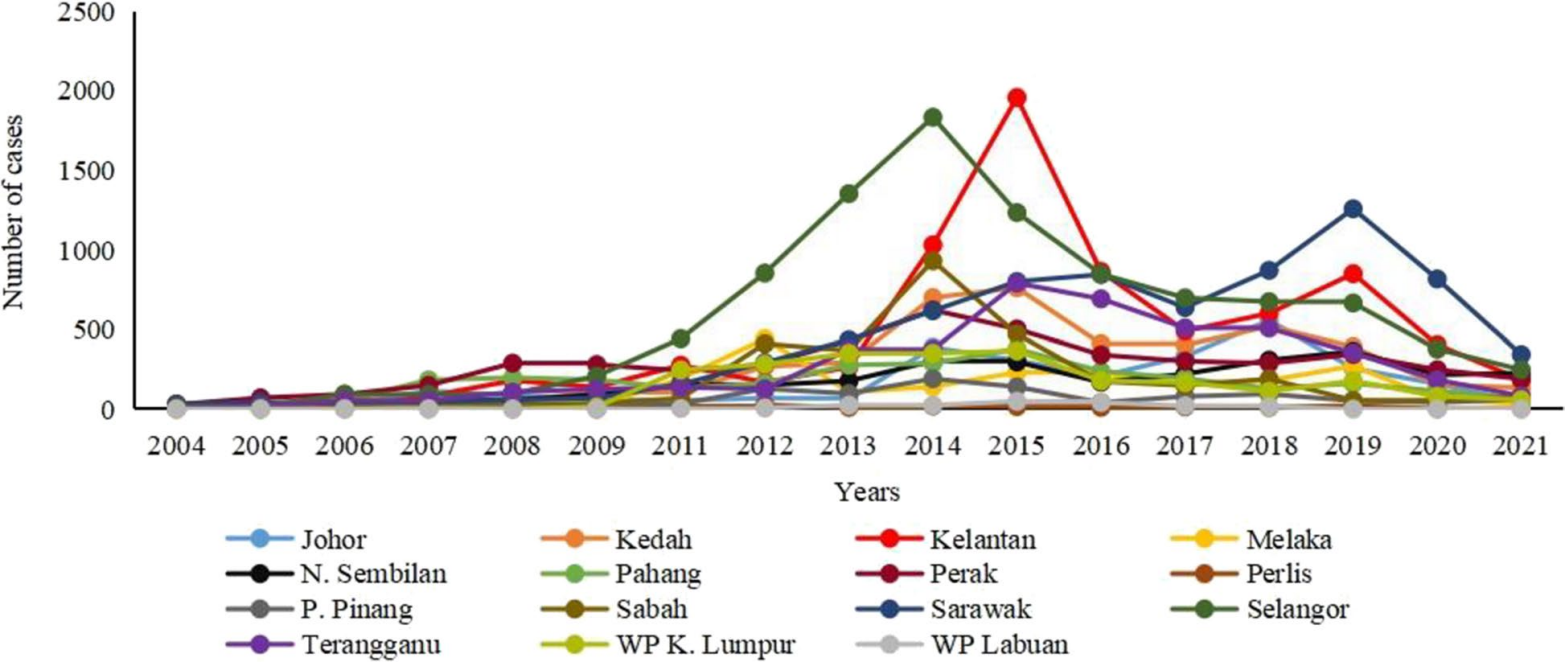
Number of cases in each state of Malaysia from 2004 to 2021. Yearly distribution of leptospirosis cases in each state of Malaysia from 2004 to 2021. Data from 2010 was not available. Source: data.gov.my

Among all states in Malaysia, Selangor, Kelantan, and Sarawak recorded the higher number of leptospirosis (Fig. 2). In 2014, 2015, and 2019, leptospirosis peaked in Selangor, Kelantan, and Sarawak, respectively. These contributed to the peaks in the year-wise distribution of leptospirosis cases in Malaysia. Perak state and the Federal Territory of Labuan recorded fewer cases throughout the 17 years.

## Potential reservoirs of *Leptospira spp*

### Animals

*Leptospira* can be isolated from a broad spectrum of animal hosts, but the primary hosts are wild animals such as rodents. From 2000 to 2022, 21 studies have been conducted in Malaysia to detect leptospires in wild and domestic animals using serological, molecular, and/or culture techniques (Table 1; Additional file 1). Detection of leptospires in wild animals (rodents, shrew, orang utan) was limited to those found in recreational spots [40–43], urban areas including human settlements and wet markets [40, 44–47], sub-urban areas [40, 48, 49], agricultural regions such as oil-palm plantations [48] and paddy fields [50], conservation center [51], and National Service Training Center [52] (Table 1). The prevalence rate of leptospires in rodents varies from 5.6 to 72% (median = 14.3%), with the highest rate detected in rodents of wet markets in Kelantan [44]. *Leptospira* was also detected in domestic animals such as dogs [53–57], cats [58], cattle [59, 60], and swine [57], as well as goat and sheep [59]. The prevalence rate of leptospires in these domestic animals ranged from 3.1 to 81.7% (median = 15.2%), with high prevalence occurring in cattle. The detection of *Leptospira* in both wild and domestic animals indicates the prevalence of *Leptospira* circulating in animals which infest the areas related to human activities as well as pets and farm animals, increasing the risk of leptospires transmission to humans in Malaysia.

**Table 1:** Detection of leptospires in animals and environments according to settings in Malaysia

**Urban areas**	**Semi-urbans**	**Agricultural regions**	**Recreational spots**	**Other areas**
Rodents [40, 44–47]; shrew [40]	Rodents [40, 48, 49]	Rodents [48, 50]	Rodents [40–43] Neela et al., 2019	Orang Utan (conservation center) [51] Rat (National Service Training Centre) [52]
Water and soil [47, 61]		Water and soil [50, 61]	Water and soil [43, 62, 63] Soil [64]	Water and soil (conservation center) [51]; Residential areas of patients [65] (National Service Training Centre) [51, 66]

### Environments

Water and soil sampling provides essential information that enhances our understanding of the leptospiral human–environment–animal relationship. Ten studies have described the distribution of *Leptospira* in the environment (Table 1; Additional file 1). The study sites included residential areas of patients with leptospirosis [65], recreational spots [43, 62–64], urban areas [47], the market [64], the National Service Training Centre [66], the Conservation Centre [51], and the agricultural regions such as rice fields, oil palm, and rubber plantations [61]. Pathogenic, intermediate, and saprophyte *Leptospira* species had been detected and isolated from water and soils in these environments in the 5–33.60% (median = 15%) of the samples. The presence of leptospires in each of the studied settings shows that leptospires occupy a range of ecological niches in Malaysia.

## Leptospira diversity

Currently, 66 species of *Leptospira* have been identified globally [67–70]. Several of these *Leptospira* species have been identified to be circulating among wild and domestic animals in Malaysia. The identified species are *L. interrogans*, *L. borgpetersenii*, *L. kirschneri*, *L. kmetyi*, *L. wolffii*, *L. weilii*, *L. noguchii*, *L. meyeri*, and *L. biflexa* [40, 41, 43–47, 50, 51, 54, 58]. In the environments, the *Leptospira* species that had been detected were *L. interrogans*, *L. borgpetersenii*, *L. wolffii*, *L. kmetyi*, *L. noguchii*, *L. meyeri*, *L. biflexa*, *L. licerasiae*, *L. fainei*, *L. inadai*, *L. alstonii*, *L. congkakensis*, *L. idonii*, *L. broomii*, *L. barantonii*, *L. putramalaysiae*, and *L. yanagawae* [43, 47, 50, 51, 63–65]*.* Several novel intermediate and saprophytic *Leptospira* species have also been isolated in the environments in Malaysia such as *L. semungkisensis*, *L. fletcheri*, *L. langatensis*, *L. selangorensis*, *L. jelokensis*, *L. perdikensis*, and *L. congkakensis* [69]. In humans, *L. weilii*, *L. kirschneri*, *L. wolffii*, and *L. interrogans* [71–74] were detected.

New serovars are also continuously being discovered. A serovar known as Malaysia was isolated from the soil samples of Johor state in 2009 [75]. Other than that, serovars Melaka (prototype strain: IMR LEP 1), Terengganu (IMR LEP 115), Sarawak (IMR LEP 175), Hardjobovis (IMR LEP 27), and Copenhagani (IMR LEP 803/11) were also isolated (Institute for Medical Research, Malaysia). Seroprevalence studies in humans and animals revealed the circulation of local serovars (Sarawak, Melaka, Terrenganu) and serovars isolated from other countries such as Autumnalis, Javanica, Bataviae, Icterohaemorrhagiae, Australis, Canicola, Ballum, Pyrogenes, Celledoni, Panama, Tarassovi, Shermani, Hebdomadis, Grippotyphosa, Hardjo, Pomona, Cynopteri, Patoc, Djasiman, Sejroe, Hardjoprajitno, and Bratislava [4, 43, 44, 49, 52–71, 76–81]. The most frequently (1.0–82.1%) detected serovars in these studies were Hardjo-bovis, Hebdomadis, Pomona, Icterohaemorrhagie, Ballum, Bataviae, Javanica, Grippotyphosa, Autumnalis, Sarawak, Patoc, Djasiman, Shermani, Pomona, and Sejroe. There has been a continuous detection and isolation of new *Leptospira* species, serovars, and strains in Malaysia since leptospirosis was reported in 1925. The recent detection of novel *Leptospira* species indicates that there could be more new *Leptospira* species, serovars, and strains as the locations and hosts studied are scarce. The detection of similar *Leptospira* species and serovars in animals, environments, and humans (Table 2) supports the transmission of leptospires from animals and environments to humans.

**Table 2:** Leptospires species and serovars detected and isolated in animals, environment, and humans in Malaysia

Animals			Environment	Humans
**Wild animals**	**Pet animals**	**Farm animals**		
L. *interrogans* *L. borgpetersenii* *L. kirschneri* *L. kmetyi* *L. wolffii* *L. weilii* *L. noguchii* *L. meyeri*	*L. interrogans* *L. borgpetersenii* *L. kirschneri* *L. kmetyi* *L. biflexa*	*L. interrogans*	*L. interrogans* *L. borgpetersenii* *L. wolffii* *L. kmetyi* *L. noguchii* *L. meyeri* *L. biflexa* *L. licerasiae* *L. fainei* *L. inadai* *L. alstonii* *L. congkakensis* *L. idonii* *L. broomii* *L. barantonii* *L. putramalaysiae* *L. yanagawae* *L. semungkisensis L. fletcheri* *L. langatensis* *L. selangorensis* *L. jelokensis* *L. perdikensis*	*L. interrogans* *L. kirschneri* *L. wolffii* *L. weilii*
Autumnalis Javanica Bataviae Icterohaemorrhagiae Australis Canicola Copenhageni Ballum Pyrogenes Celledoni Panama Tarassovi Shermani Hebdomadis	Autumnalis Javanica Bataviae Icterohaemorrhagiae Australis Canicola Copenhageni Ballum Pyrogenes Celledoni Grippotyphosa Lai Hardjobovis Hardjo Pomona Bratislava	Autumnalis Javanica Bataviae Icterohaemorrhagiae Australis Canicola Copenhageni Ballum Pyrogenes Tarassovi Hebdomadis Grippotyphosa Lai Hardjobovis Hardjo Pomona Malaysia Cynopteri Sarawak Patoc Melaka Terengganu Djasiman Sejroe Hardjoprajitno	Hebdomadis	Autumnalis Javanica Bataviae Icterohaemorrhagiae Australis Canicola Copenhageni Ballum Pyrogenes Celledoni Panama Tarassovi Shermani Hebdomadis Grippotyphosa Lai Hardjobovis Hardjo Pomona Malaysia Cynopteri Sarawak Patoc Melaka Terengganu Djasiman Sejroe Hardjoprajitno

## Risk factors

**Fig. 3:**
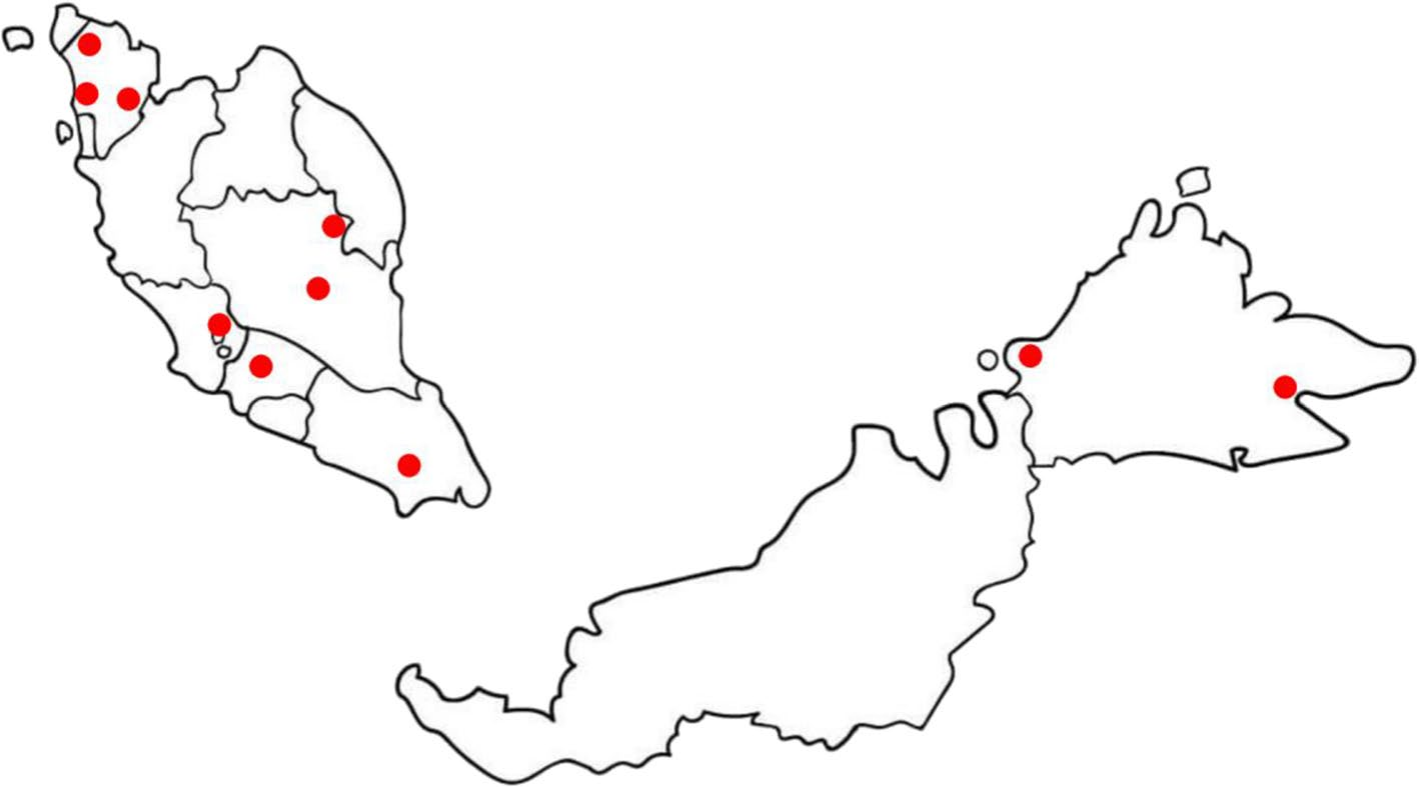
Death from leptospirosis caused by water activities. The symbols indicate locations where people died of leptospirosis due to involvement with recreational and water activities during 2000–2020

Heavy rainfall and flood are well-known risk factors for leptospirosis [82]. Flood is one of the contributing factors that led to the high number of leptospirosis cases in Kelantan at the end of 2014 and early 2015 [83]. From 2010 to 2020, several local newspapers reported human deaths from leptospirosis as a result of recreational water activities such as swimming in different localities in Malaysia [84–90] (Fig. 3). It was also reported in 2010 that six people died of leptospirosis after a search and rescue operation in Hutan Lipur, Lubuk Yu, and Pahang state [91]. A few people also experienced severe leptospirosis after swimming in a waterfall [92] and kayaking in a river [93]. In Sarawak, the weekly washing of clothes in local rivers was associated with leptospirosis among hospital patients [73]. Among wet market workers in two locations were 33.6% [76] and 46.3% [78]. In Sabah, two outbreaks were reported in 2000 [71] and 2004 [94], which were related to water activities such as kayaking and swimming in Segama River and swimming in the creek near an oil palm plantation in Beaufort, respectively.

Occupations such as urban sweepers, landscapers, garbage collectors, and lorry drivers are also risk factors for leptospirosis [95]. One of the major industries in Malaysia is the palm oil sector, and the seroprevalence of *Leptospira* among these workers is 28.6% [79]. Likewise, workers in animal farms also have a high (72.5%) seroprevalence for leptospires [77]. Owning pets such as dogs and cats increases the likelihood of the owner in acquiring leptospirosis [53–58]. The social conditions, such as poor urban communities, also contribute to the incidence of leptospirosis [96]. A number of risk factors have been identified to be associated with leptospirosis in Malaysia with activities involving waters contributing the most to the occurrence of leptospirosis.

## Level of leptospirosis knowledge

The knowledge and attitude towards leptospirosis influence individuals’ behavior in practicing preventive measures against this infection. Hence, assessing people’s knowledge, attitude, and practices (KAP) toward leptospirosis is crucial. This information is critical to developing strategies for behavior changes toward safer practices. Several studies have been conducted in different communities and workplaces in Malaysia between 2018 and 2022. Such a study conducted in rural communities in Selangor showed that more than half of the participants had poor knowledge, and it was associated with unacceptable attitudes and practices towards leptospirosis [97]. In urban communities in the same locality, the majority of the participants (> 80%) had poor knowledge and practices on leptospirosis prevention despite having a positive attitude toward waste management [98]. In another study conducted in urban and rural communities in northeastern Malaysia, more than half of the participants had good knowledge and a positive attitude towards leptospirosis [99]. However, the level of knowledge and attitude were insufficient to translate into good behavior practices. A study on visitors to a recreational forest in Terengganu state showed higher knowledge, positive attitudes, and good practices toward preventing leptospirosis [100]. However, most of them still had limited knowledge of the route of transmission and prevention measures. In the workplace environment, studies conducted in northeastern Malaysia on town service workers [101] and army personnel [102], as well as dog handlers [103] showed that the majority of them had a relatively low level of KAP toward leptospirosis. Another study in agricultural communities showed that despite their good attitude towards the infection and moderate knowledge, they had unsatisfactory practices preventing leptospirosis [61]. Overall, these studies show that public awareness toward leptospirosis is still low in Malaysia.

## Discussion

Leptospirosis is endemic in Malaysia, and the cases increased from 2004 to 2015, with the peak in 2015, then decreasing till 2017, and increasing till 2019. The decline in cases in 2020 and 2021 was presumably associated with behavioral changes due to the MCO. As the MCO has been lifted, it is expected that the cases might increase again as more people are involved in outdoor and recreational activities. Recently, local newspapers reported that a prisoner died [104] and a boy had a coma due to leptospirosis [105].

This review compiled the studies of leptospirosis in Malaysia focusing on the isolation and detection of leptospires in animals, environment, and humans as well as leptospirosis cases reported in the local newspapers. Most of the studies performed independent research which either focused on animals or environment or humans (Additional file 1). Only one study [43] included animals, environment, and humans. However, this study used a different method of detection of leptospires in animals (culture and PCR), environment (culture and PCR), and humans (MAT), and the species identified in animals and environment differed. Three studies [47, 50, 51] included both animals and environments and detected similar *Leptospira* species. One study [55] included both domestic animals and humans and detected similar serovars. Based on these few studies, it might be difficult to establish a direct transmission of similar genetic profiles of infecting *Leptospira* species or strains between animals, environments, and humans. Nevertheless, it still can be concluded that the detection of similar *Leptospira* species and serovars in human, animal, and environmental samples showed human-animal-environment interconnection. *L. interrogans* could be the predominant circulating species as it is detected in rodents infesting a range of ecological settings as well as in domestic animals, environments, and humans. *L. interrogans* was also the earliest detected *Leptospira* species in Malaysia [9]. Autumnalis, Javanica, Bataviae, Icterohaemorrhagiae, Australis, Canicola, Copenhageni, Ballum, and Pyrogenes are the predominant serovars as these serovars were detected in both wild and domestic animals and humans.

Based on the risk factors, leptospirosis cases, and death are mainly associated with recreational and non-recreational water activities. In other countries such as Brazil and Thailand, the occurrence of leptospirosis is largely associated with animal farming and agriculture [106, 107]. The low public’s awareness toward leptospirosis might be also one of the contributing factors to the likelihood of leptospirosis infections in humans in Malaysia.

Although previous studies provided insight into the human-animal-environment relationship in the occurrence of leptospirosis, more is needed to provide a complete understanding and knowledge of the epidemiology of leptospirosis in Malaysia as the studies were only performed in a limited number of settings and states. Hence, more studies on humans, animals, and environments are needed covering various locations in Malaysia. In other countries, *Leptospira* has also been isolated from “unconventional” hosts such as elephants, porcupines, bats, snakes, and frogs [108]. It would be comprehensive to determine “unconventional” hosts in Malaysia to understand the epidemiology of leptospirosis as these animals are abundant in this country.

It is of paramount importance not only to identify but also to isolate the local *Leptospira* species, serovars, and strains. The current gold standard, MAT, for diagnosing leptospirosis requires a panel of locally prevalent serovars. Local serovars such as Sarawak and Terengganu have been detected in humans [4, 76, 79] and cattle [60]. Therefore, the inclusion of local serovars can avoid the possibility of misdiagnosis. Only a few serovars have been isolated in Malaysia, which are restricted to a few locations. Hence, more studies are needed to identify and isolate locally circulating new serovars, especially in states where leptospirosis study is scarce, such as Sabah.

Furthermore, the *Leptospira* species, serovars, and strains have different virulence levels and are associated with a broad spectrum of clinical presentations. It is worth noting that some people who died of leptospirosis in Malaysia had pulmonary hemorrhage [93], coughing of blood, and breathing difficulty [93]. Leptospirosis with pulmonary hemorrhage was also observed in patients and returned travelers from Malaysia [109–112]. The infecting *Leptospira* species or serovars are not known for most of these patients except for one case caused by serovar Lai: Langkawi [107], showing that the leptospires circulating in Malaysia can cause severe disease with hemorrhagic manifestations. In vivo, a study in an animal model showed that the local *L. interrogans* can cause severe infection and pulmonary hemorrhage in hamsters [113]. Hence, identification and isolation of circulating *Leptospira* from animals, environments, and humans in Malaysia are necessary to understand the virulence potential of this bacteria.

The limited studies in assessing public awareness toward leptospirosis urge the need to do more studies in different communities, workplaces, and localities are needed to assess the overall level of knowledge and awareness regarding leptospirosis in Malaysia.

KAP studies have been performed only in Peninsular Malaysia, and conducting similar studies in Sabah and Sarawak, which recorded many leptospiroses, is paramount. Nevertheless, previous studies also showed that more campaigns are needed to generate awareness of leptospirosis and dissemination of information.

## Conclusion

Leptospirosis is endemic in Malaysia. This review highlighted the need to perform more studies on leptospires in animals and environments in different places for prevention strategies and improving diagnosis and early treatment. Assessing people’s awareness of leptospirosis is needed to implement the strategy that could help them perform good practices toward leptospirosis prevention.

### Supplementary Information

**Additional file 1: **Summary of studies on animals, environment and humans in Malaysia.

## Data Availability

Not applicable.

## Abbreviations

MAT: Microscopic agglutination test
PCR: Polymerase chain reaction
MCO: Movement control order
CFR: Case fatality rates
KAP: Knowledge, attitude, and practices
