# Supplementary material for: Leptospirosis in Malaysia: current status, insights, and future prospects
Source: J Physiol Anthropol. 2023 Dec 12;42:30. doi: 10.1186/s40101-023-00347-y (PMC10714552; doi:10.1186/s40101-023-00347-y)
Supplement: Supplementary file 1 — Additional file 1: Summary of studies on animals, environment and humans in Malaysia. [file 40101_2023_347_MOESM1_ESM.docx]

**Additional File 1: Summary of studies on animals, environment and humans in Malaysia**

| **No** | **Reservoir/Host** | **Setting** | **Location** | **% of positivity** | **Method of detection** | **Species/serovars** | **Reference** |
| --- | --- | --- | --- | --- | --- | --- | --- |
|  | Rodents and shrew | Human settlements, recreactional spots | Selangor | 14.3% | PCR | *L. interrogans, L. borgpetersenii, L. kirschneri, L. weilii* | [40] |
|  | Small mammals  (Rat, shrew, squirrel) | Recreational forest | Terengganu | 20% | PCR | *L. interrogans* | [41] |
|  | Rodents | Recreational spots | Johor | 13% | PCR | NA | [42] |
|  | Rats | Recreational areas | Selangor | 50% | Culture, PCR | *L. interrogans, L. borgpetersenii* | [43] |
|  | Rats | Urban  (wet markets) | Kelantan | 72% | PCR | *L. interrogans, L. borgpetersenii* | [44] |
|  | Rats | Urban  (wet markets and residential area) | Kuala Lumpur | 6.7% | Culture | 1. *borgpetersenii*serovar Javanica,   *L. interrogans* serovar Bataviae | [45] |
|  | Rodents | Urban, developing, rural | Sarawak | 31.6% | PCR | *L. interoggans, L. borgpetersenii* | [46] |
|  | Rats | Urban areas  (public university, villages, residential areas, commercial centres, hawker centres, markets) | Sarawak | Rat 5.6% | PCR | *L. interrogans,*  *L. noguchii, L. meyeri* | [47] |
|  | Rats | National Service Training Camps; Oil palm estates;  Royal Belum Rainforest;  Suburban areas;  PULAPOL | Kelantan, Terengganu, Malacca, Perak, Negeri Sembilan and Selangor | 12.3% | Culture, PCR | Pathogenic *Leptospira* | [48] |
|  | Rodents | Sub-urbans  (Near to residential areas) | Sarawak | 40.7% | MAT | Autumnalis, Bataviae, Hebdomadis, Pyrogenes, Canicola, Australis, Copenhageni, Panama, Icterohaemorrhagiae, Ballum, Tarassovi, Shermani,Celledoni | [49] |
|  | Rats | Paddy fields | Sarawak | 3.2% | PCR | Intermediate *Leptospira* | [50] |
|  | Orang Utan  Rodents | Conservation Centre | Perak | Orang Utan 17.6%  Rodents 4.3% | PCR | Orang Utan: *L. wolffii, L. kmetyi*  Rodents: *L. wolffii* | [51] |
|  | Rats | National Service Training Centres | Kelantan and Terengganu | Kelantan: 17.3%  Terengganu: 18.4% | MAT | Icterohaemorrhagiae, Canicola, Ballum, Pyrogenes, Hebdomadis | [52] |
|  | Dogs | Healthy dogs and dogs with kidney disease | Selangor | 7.0% | MAT, PCR | Canicola, Icterohaemorrhagiae | [53] |
| 15. | Dogs with kidney & liver disease | Veterinary hospital & clinic  (pet dogs) | Selangor | MAT 42.7%,  PCR 42.7% | MAT, PCR | Serovars (Bataviae, Javanica, Icterohaemorrhagiae, Australis)  Species (*L. interrogans, L. borgpetersenii, L. kirschneri, L. kmetyi*) | [54] |
| 16. | Dogs | Four working dog organisations and shelters | Johor, Kuala Lumpur, Negeri Sembilan, Selangor | 26.3%, | MAT | Icterohaemorrhagiae, Canicola, Grippotyphosa, Australis, Autumnalis, Lai, Ballum, Hardjobovis, Hardjo, Bataviae, Javanica, Pyrogenes, Copenhageni | [55] |
| 17. | Dogs (Vaccinated dogs) | Canine unit from different government agencies | - | 3.1% | MAT | Autralis, Bataviae, Javanica | [56] |
| 18. | Stray dogs,  Swine | Swine farms, stray cat and dogs | Selangor | Stray dogs: 7.3%  Swine: 6% | PCR, MAT | *L. interrogans* (Canicola, Icterohaemorrhagiae, Pomona, Bratislava) | [57] |
| 19. | Cat | Shelter cat | NA | 29.2% | MAT, PCR, culture | Species *(L. interrogans, L. biflexa)*  Serovar (Bataviae, Javanica, Ballum) | [58] |
| 20. | Cattle, goats, sheep | Livestock farm (flood-prone areas) | Kelantan | 11.75% | MAT | Hardjobovis, Hebdomadis, Pomona, Malaysia, Celledoni, Tarrasovi, Pyrogenes, Australis, Grippothyposa, Cynopteri, Canicola, Icterohaemorrhagiae, Bataviae, Lai, Javanica, Autumnalis, Ballum | [59] |
| 21. | Cattle | Cattle farms | Kelantan | 81.7% | MAT | Sarawak, Patoc, Hardjobovis, Australis, Hardjoprajitno, Melaka, Terengganu, Tarrasovi, Pomona, Copenhageni, Grippotyphosa, Autumnalis, Lai, Bataviae, Pyrogenes, Canicola, Djasiman, Icterohaemorrhagiae | [60] |
| 1. | Water and soil | Recreational areas | Selangor | 38.9% | Culture, PCR | *L. kmetyi, L. wolffii* | [43] |
| 2. | Water and soil | Urban areas  (public university, villages, residential areas, commercial centres, hawker centres, markets) | Sarawak | Water: 1.9%  Soils: 11.6%; | PCR | *L. interrogans, L. borgpetersenii*  *L. noguchii, L. wolffii, L. meyeri, L. inadai* | [47] |
| 3. | Water and soil | National Service Training Centres,  Paddy fields | Sarawak | Water: 2.9% (pathogenic *Leptospira*),  1.9% (intermediate *Leptospira*), 3.3% (saprophytic *Leptospira*)  Soil: 2.9% (pathogenic *Leptospira*)  0.5% (intermediate *Leptospira*) 0.5% (saprophytic *Leptospira*) | Culture, PCR | Pathogenic, intermediate, saprophytic *Leptospira* | [50] |
| 4. | Water and soil | Conservation Centre | Perak | Water: 18.9%  Soil: 17.9% | Culture, PCR | *L. yanagawae*  *L. meyeri*  *L. idonii*  *L. wolffii*  *L. Interrogans*  *L.kmetyi* | [51] |
| 5. | Water and soil | Residential areas  (Confirmed leptospirosis patients’ residences) | Kelantan | 42.8% | Culture | *L. kmetyi,  L. wolffii, L. licerasiae, L. fainei, L. inadai , L. meyeri* | [61] |
| 6. | Water and soil | Environment (amenity forest) | Perak | 13% | PCR | Pathogenic *Leptospira* | [62] |
| 7. | Water and soil | Recreational areas | Peninsular Malaysia | 33.6% | Culture | *L. biflexa, L. congkakensis, L. idonii, L. broomii, L. licerasiae, L. wolffii, L. kmetyi, L. barantonii, L. putramalaysiae* | [63] |
| 8. | Water and soil | Environment (Market and recreational area) | Kelantan | 22.9% | Culture | *L. wolffii, L. licerasiae, L. meyeri, L. alstonii* | [64] |
| 9. | Water and soil | National Service Training Centres | Kelantan, Terengganu | 10.34% | Culture | Pathogenic *Leptospira,*  Hebdomadis | [65] |
| 10. | Water and soil | Rice field,  Oil palm plantations | Terengganu | 5% | PCR | NA | [66] |
| 1. | Human  (Seroprevalence) | Urban sanitation workers | Sabah | 43.8% | MAT, PCR | Patoc, Sarawak, Terengganu, Copenhageni, Hardjobovis, Javanica, Lai, Icterohaemorrhagiae | [4] |
| 2. | Human  (Patients with clinical leptospirosis) | Reserve military recruits | Selangor | 16.6% | MAT | Autumnalis, hardjobovis | [43] |
| 3. | Human  (Seroprevalence) | Four working dog organisations and shelters | Johor, Kuala Lumpur, Negeri Sembilan, Selangor | 34.5% | MAT | Grippotyphosa, Icterohaemorrhagiae, Canicola, Australis, Hardjo, Bataviae, Pyrogenes, Hebdomadis, Patoc, Cynopteri, Malaysia | [55] |
| 4. | Human  (Patients with clinical leptospirosis) | Eco-challenge multisport | Segama, Sabah | 77% | MAT positive from ELISA positive | *L. weilii,* Australis, Hebdomadis | [71] |
| 5. | Humans  (Patients with clinical leptospirosis) | Hospitals | Selangor, Perak | 56% | MAT, PCR | *L. interrogans, L. kirschneri, L. wolffii* | [72] |
| 6. | Human  (Patients with clinical leptospirosis) | Hospitals | Sarawak | 37.40% | qPCR, rapid test & ELISA | *L. interrogans* | [73] |
| 7. | Human  (Patients presumptive diagnosis of dengue-like illness) | District health clinics | Selangor | 4.7% | PCR | *L. interrogans* | [74] |
| 8. | Human  (Seroprevalence) | Wet markets workers | Kelantan | 33.6% | MAT | Melaka (IMR LEP 1), Terengganu (IMR LEP 115), Sarawak (IMR LEP 175), Copenhageni (IMR LEP 803/11), Hardjobovis (IMR LEP 27), Australis, Autumnalis, Bataviae, Canicola, Grippotyphosa, Hardjoprajitno, Icterohaemorrhagiae, Javanica, Pyrogenes, Tarrasovi, Djasiman, Patoc and Pomona | [76] |
| 9. | Human  (Seroprevalence) | Cattle farmers | Kelantan | 72.5% | MAT | Sarawak, Patoc, Hardjobovis | [77] |
| 10. | Human  (Seroprevalence) | Wet markerts and food premises | Selangor | 46.30% | MAT | LEP175, LEP115, LEP27, Patoc, Australis, Grippotyphosa | [78] |
| 11. | Human  (Seroprevalence) | Workers in plantation (oil palm) | Melaka, Johor | 28.6% | MAT | Sarawak, Patoc, Celledoni, Javanica, Australis, Autumnalis, Pyrogenes, Copenhageni, Terengganu | [79] |
| 12. | Humans  (Seroprevalence) | Rural communities | Sarawak | 37.4% | MAT, ELISA | Djasiman, Shermani, Pomona, Australis, Autumnalis, Bataviae, Canicola, Celledoni, Grippotyphosa, Hardjobovis, Hebdomadis, Icterohaemorrhagiae, Javanica, Pyrogenes, Patoc, Ballum, Copenhageni, Panama, Tarassovi, IMR175 | [80] |
| 13. | Humans  (Seroprevalence) | Febrile cases | 10 hospitals in northeastern Malaysia | 8.4% | MAT | Sejroe, Pyrogenes, Icterohaemorrhagiae, Celledoni, Javanica, Australis, Bataviae, Autumnalis | [81] |
| 14. | Human  (Seroprevalence) | PPR - urban poor communities | Kuala Lumpur | 12.6% | ELISA | NA | [96] |
